# Supplementary material for: P-N Junction-Based Si Biochips with Ring Electrodes for Novel Biosensing Applications
Source: Biosensors (Basel). 2019 Oct 11;9(4):120. doi: 10.3390/bios9040120 (PMC6956254; doi:10.3390/bios9040120)
Supplement: Supplementary file 1 [file biosensors-09-00120-s001.docx]

**Table S1.** The impedance magnitude for boron doped biochip BS5 with different amount of analyte (1 – 5 µl *Lysinibacillus sphaericus* JG-A12) at frequencies 40 Hz, 400 Hz, and 4 kHz. This table is corresponding to the experimental results in Fig. 8a.

|  | **\|Z\|( Ω) @ 40 Hz** | **\|Z\|( Ω) @ 400 Hz** | **\|Z\|( Ω) @ 4000 Hz** |
| --- | --- | --- | --- |
| **BS5+W20 +B1** | 19294 | 18586 | 9218 |
| **BS5+W20 +B2** | 19176 | 18357 | 9183 |
| **BS5+W20 +B3** | 18800 | 17919 | 9109 |
| **BS5+W20 +B4** | 18554 | 17573 | 9053 |
| **BS5+W20 +B5** | 18288 | 17240 | 8361 |

^W20 = 20µl DI water, B1 = 1µl bacteria, B2 = 2µl bacteria, B3 = 3µl bacteria, B4 = 4µl bacteria, B5 = 5µl bacteria^

**Table S2.** The impedance magnitude for the biochip PS5 with different amount of analyte (1 – 5 µl *Lysinibacillus sphaericus* JG-A12) at frequencies 40 Hz, 400 Hz, and 4 kHz. This table is corresponding to the experimental results in Fig. 8b.

|  | **\|Z\|( Ω) @ 40 Hz** | **\|Z\|( Ω) @ 400 Hz** | **\|Z\|( Ω) @ 4000 Hz** |
| --- | --- | --- | --- |
| **PS5+W20 +B1** | 1219 | 1146 | 1032 |
| **PS5+W20 +B2** | 1188 | 1109 | 989 |
| **PS5+W20 +B3** | 1171 | 1111 | 972 |
| **PS5+W20 +B4** | 1158 | 1040 | 899 |
| **PS5+W20 +B5** | 1137 | 1024 | 856 |

^W20 = 20µl DI water, B1 = 1µl bacteria, B2 = 2µl bacteria, B3 = 3µl bacteria, B4 = 4µl bacteria, B5 = 5µl bacteria^
